# *Epinephelusrankini* Whitley, 1945, a valid species of grouper (Teleostei, Perciformes, Epinephelidae) from Western Australia and southeast Indonesia

**DOI:** 10.3897/BDJ.10.e90472

**Published:** 2022-10-14

**Authors:** Xiaoying Cao, Haohao Wu, Haoran Zhang, Lina Wu, Shaoxiong Ding

**Affiliations:** 1 Xiamen Key Laboratory of Urban Sea Ecological Conservation and Restoration, College of Ocean and Earth Sciences, Xiamen University, Xiamen, China Xiamen Key Laboratory of Urban Sea Ecological Conservation and Restoration, College of Ocean and Earth Sciences, Xiamen University Xiamen China; 2 Function Laboratory for Marine Fisheries Science and Food Production Processes, Qingdao National Laboratory for Marine Science and Technology, Qingdao, China Function Laboratory for Marine Fisheries Science and Food Production Processes, Qingdao National Laboratory for Marine Science and Technology Qingdao China

**Keywords:** *Epinephelusmultinotatus*, morphology, mtDNA, taxonomy, validity

## Abstract

**Background:**

The grouper *Epinephelusrankini*, described from the waters off Western Australia, has long been regarded as a junior synonym of *Epinephelusmultinotatus*. However, the two species are discernible as distinct species on the basis of their morphological characteristics and genetic differences by the holotype material and non-type of specimens.

**New information:**

In this study, *Epinephelusrankini* is considered as a valid species and re-described based on the examination of the holotype and additional specimens. *Epinephelusrankini* can be distinguished from the closely-related species *E.multinotatus* by the following combination of characters: body dark greyish-brown to chocolate with irregular white blotches (vs. body pale brownish-grey with irregular and small white blotches in *E.multinotatus*), absence of small dark brown spots (vs. numerous small dark brown spots in *E.multinotatus*). Furthermore, genetic differences between the two species strongly support the validity of both species based on molecular analysis (mtDNA, COI gene). In addition based on the sampling range, *E.rankini* was observed range from the Abrolhos Islands of Western Australia to south-eastern Indonesia, while *E.multinotatus* ranges from the Persian Gulf to southern Mozambique.

## Introduction

The family Epinephelidae, known as groupers, comprises more than 170 species in 16 genera (Craig et al. 2011, Liu et al. 2013, Nelson et al. 2016, Frable et al. 2018, Wu et al. 2020). The genus *Epinephelus* Bloch, 1793 (Perciformes, Epinephelidae) is the most diverse, containing approximately 90 valid species (Craig et al. 2011). Groupers widely inhabit coral reefs and rocky coastlines of the tropics and subtropical seas and are usually at the top of the food webs (Ding et al. 2018). Due to the important commercial and ecological value of groupers, many studies have been carried out on their taxonomy and phylogenetic relationship (Ma et al. 2016, Ma and Craig 2018). However, because of the species richness, wide distribution range and variable body colour of groupers, earlier classification may be problematic. In recent years, with increases in the sampling range, sampling amount and the emergence of molecular technology, some grouper classifications have undergone new changes, such as *Epinephelusquinquefasciatus* (Bocourt, 1868) (Craig et al. 2009), *Epinephelusgeoffroyi* (Klunzinger, 1870) (Randall et al. 2013), *Epinephelusmoara* (Temminck & Schlegel, 1842) (Liu et al. 2013) being recovered as valid species and *Epinepheluskupangensis* (Tucker, Kurniasih & Craig, 2016), *Epinepheluscraigi* (Frable, Tucker & Walker, 2018), *Epinephelustankahkeei* (Wu, Qu, Lin, Tang & Ding, 2020) and others being identified as new species (Tucker et al. 2016,Frable et al. 2018,Wu et al. 2020). This research provides the most recent material for understanding grouper taxonomy and phylogenetics and implies the possibility of more cryptic species under the current taxonomic system of groupers.

*Epinephelusrankini* Whitley, 1945 was first collected by Mr. F. J. Rankin in the Onslow, Western Australia in late 1944 and then described by Whitley (1945), but it has long been considered a junior synonym of the white-blotched grouper *Epinephelusmultinotatus* (Peters 1876；type locality: Mauritius) (Polovina and Ralston 1987, Hutchins and Smith 1991). Between 2019 and 2021, we collected two different morphotypes of white-blotched grouper from Western Australia and the western Indian Ocean. After careful examination of the holotypes of *E.multinotatus* and *E.rankini* and non-type specimens, we found that *E.multinotatus* and *E.rankini* differed significantly in body colour patterns, which supported them as two distinct species. The genetic differences [mitochondrial DNA of the barcode region, cytochrome c oxidase subunit I (COI) gene] between these two species also strongly support this conclusion. In this paper, we describe the morphological and genetic differences between *E.multinotatus* and *E.rankini* and re-describe *E.rankini* and *E.multinotatus* as well.

## Materials and methods

### Fish collection and morphological measurement

Specimens from western Indian Ocean, Western Australia and southeast Indonesia were examined, including the holotypes (Fig. 1) of *E.multinotatus* and *E.rankini*. Specimens of *E.rankini* from Indonesia and the type locality, as well as specimens of *E.multinotatus* from Maldives, Seychelles, Africa and Mauritius (type locality) were also examined. The sampling information is listed in Suppl. materials 1, 2 and the collection site map was generated by ODV v.5.1.5 software (Schlitzer 2002). New specimens were purchased from the fish market and preserved in anhydrous ethanol and deposited in the Fish Collection of the College of Ocean and Earth Sciences, Xiamen University. Institutional acronyms followed Sabaj (2016). The counts and measurements of specimens were taken following Heemstra and Randall (1993), using a digital caliper or rule to measure. Measurements of the *E.rankini* and *E.multinotatus* as percentages of standard length are listed in Table 1.

### DNA extraction and PCR amplification

Total DNA of *E.rankini*, *E.multinotatus* and closely-related species was isolated from fresh dorsal-fin rays using a standard phenol-chloroform protocol and the ethanol precipitation method and then preserved at -20°C. Partial sequences of the mitochondrial *COI* were amplified using a pair of primers (Fish F1, 5’-TCAACCAACCACAAAGACATTGGCAC-3’ and Fish R1, 5’-TAGACTTCTGGGTGGCCAAAGAATCA-3’) by polymerase chain reaction (PCR) (Ward et al. 2005). PCR reactions of 25 µl were performed following manufacturer's instructions with Taq DNA Polymerase, template DNA (10-50 ng) and primers (10 pmol). PCR reactions consisted of 94℃ for 4 min, 35 cycles of 94℃ for 30 s, 55℃ for 30 s and 72℃ for 45 s and an extension at 72℃ for 10 min. Sequences were generated on an ABI 3730xl DNA analyser (Sangon Biotech, Shanghai, China) following manufacturer's instructions.

### Sequence analysis

A total of 34 COI sequences in this study were manually edited using Sequencher 5.4.6 (http://www.genecodes.com) software and then the shared 642 bp were extracted from each sequence for subsequent analysis. The intraspecific and interspecific genetic distances were calculated using the Kimura two-parameter (K2P) distance model with MEGA 11 (Kimura 1980;Tamura et al. 2021) and the cut-off distance values for the average interspecific genetic distance and average intraspecific distance were 0.01661 and 0.0034, respectively (Qu et al. 2018). The best model of sequence evolution was inferred by jModelTest 2.1.10 and the TrN+I+G model was selected based on the Akaike Information Criteria (AIC) (Darriba et al. 2012). Maximum Likelihood (ML) analysis in PhyML 3.1 with 1000 bootstrap replicates was performed in this study (Guindon and Gascuel 2003). According to the phylogenetic tree of the family Epinephelidae constructed by Ma et al. (2016), we selected *Epinepheluscyanopodus* (Richardson, 1846) and *Epinephelusflavocaeruleus* (Lacepède, 1802), which clustered with *E.multinotatus* in the same branch, for analysis together, and *Epinepheluschlorostigma* (Valenciennes, 1828) and *Epinephelusareolatus* (Forsskål, 1775), another sister branch that shares a common ancestor with *E.multinotatus*, as outgroup. The PhyML tree was analysed using the online (http://species.h-its.org/ptp/) version of the programme bPTP (Zhang et al. 2013) to generate species hypotheses for comparison. The parameters used in the analysis included: MCMC generations = 100000, thinning value = 100, burn-in value = 0.1 and other parameters were set to the default values. Moreover, Automated Barcode Gap Discovery analysis (ABGD; http://wwwabi.snv.jussieu.fr/public/abgd/; Puillandre et al. 2011) was also used to further assess species boundaries based on COI data. The parameters were Pmin = 0.001, Pmax = 0.1, setp = 10, X (relative gap width) = 1.5, Nb bins (for distance distribution) = 20 and genetic distance model = Kimura (K80). In addition, character-based DNA barcoding (CBB; Desalle et al. 2005) was performed to delimit and diagnose species by specific nucleotide combinations within shared sites (for CBB details, see Bergmann et al. 2009, Ottoni et al. 2019, Guimarães et al. 2020). Optimisation of nucleotide substitutions amongst lineages from Bayesian Inference topology used PAUP4 (Swofford 1998). Each nucleotide substitution is represented by its relative numeric position, which was determined by sequence alignment with the complete mitochondrial genome of *E.chlorostigma* (NC_032086.1: 5569-6210). The specific nucleotide substitution is presented in parentheses and the results are presented in the molecular diagnostics section and Suppl. material 3. The genetic material used in this study conforms to the Nagoya Protocol on Access to Genetic Resources and the Fair and Equitable Sharing of Benefits Arising from their Utilisation to the Convention on Biological Diversity.

## Data resources

Sample information and GenBank accession numbers for all DNA sequences in this study can be found in Suppl. material 2.

## Taxon treatments

### Epinephelus rankini

Whitley, 1945 (Figs. 1a and 2a, Table 1)

8943166D-DB2D-529F-811A-FBF8FD8361E6

9A7880BD-7DF2-4F4C-A07B-9371A83FEAF1

#### Materials

- **Type status:** Holotype. **Occurrence:** recordedBy: Whitley; **Taxon:** taxonID: WAM P.2847-001; scientificNameID: *Epinephelusrankini*; kingdom: Animalia; phylum: Chordata; class: Actinopterygii; order: Perciformes; family: Epinephelidae; genus: Epinephelus; scientificNameAuthorship: (Whitley, 1945); **Location:** locality: Western Australian; **Record Level:** language: en; institutionCode: WAM
- **Type status:** Paratype. **Taxon:** scientificNameID: *Epinephelusrankini*; kingdom: Animalia; phylum: Chordata; class: Actinopterygii; order: Perciformes; family: Epinephelidae; genus: Epinephelus; **Location:** locality: Indonesia; **Identification:** dateIdentified: 2019-4; **Record Level:** collectionID: ZMUA-epran01; institutionCode: ZMUA; collectionCode: fish
- **Type status:** Paratype. **Taxon:** scientificNameID: *Epinephelusrankini*; kingdom: Animalia; phylum: Chordata; class: Actinopterygii; order: Perciformes; family: Epinephelidae; genus: Epinephelus; **Location:** locality: Thevenard Island, Western Australia; **Identification:** dateIdentified: 2021-11; **Record Level:** collectionID: ZMUA-epran02; institutionCode: ZMUA; collectionCode: fish
- **Type status:** Paratype. **Taxon:** scientificNameID: *Epinephelusrankini*; kingdom: Animalia; phylum: Chordata; class: Actinopterygii; order: Perciformes; family: Epinephelidae; genus: Epinephelus; **Location:** locality: Dirk Hartog Islands, Western Australia; **Identification:** dateIdentified: 2021-11; **Record Level:** collectionID: ZMUA-epran03; institutionCode: ZMUA; collectionCode: fish
- **Type status:** Paratype. **Taxon:** scientificNameID: *Epinephelusrankini*; kingdom: Animalia; phylum: Chordata; class: Actinopterygii; order: Perciformes; family: Epinephelidae; genus: Epinephelus; **Location:** locality: Indonesia; **Identification:** dateIdentified: 2021-12; **Record Level:** collectionID: ZMUA-epran04; institutionCode: ZMUA; collectionCode: fish
- **Type status:** Paratype. **Taxon:** scientificNameID: *Epinephelusrankini*; kingdom: Animalia; phylum: Chordata; class: Actinopterygii; order: Perciformes; family: Epinephelidae; genus: Epinephelus; **Location:** locality: Indonesia; **Identification:** dateIdentified: 2021-11; **Event:** eventDate: 2006; **Record Level:** collectionID: ZMUA-epranA; institutionCode: ZMUA; collectionCode: fish
- **Type status:** Paratype. **Taxon:** scientificNameID: *Epinephelusrankini*; kingdom: Animalia; phylum: Chordata; class: Actinopterygii; order: Perciformes; family: Epinephelidae; genus: Epinephelus; **Location:** locality: Western Australia; **Identification:** dateIdentified: 2019-8; **Record Level:** collectionID: ZMUA-epranB; institutionCode: ZMUA; collectionCode: fish
- **Type status:** Paratype. **Taxon:** scientificNameID: *Epinephelusrankini*; kingdom: Animalia; phylum: Chordata; class: Actinopterygii; order: Perciformes; family: Epinephelidae; genus: Epinephelus; **Location:** locality: Western Australia; **Identification:** dateIdentified: 2019-8; **Record Level:** collectionID: ZMUA-epranC; institutionCode: ZMUA; collectionCode: fish

#### Description

Head large and head length 2.6 (2.6-2.8) in SL, orbit diameter 6.8 (6.5-6.9) in head; interorbital broadly convex and width 4.5 (4.1-4.5) in head. Snout length 4.08 (3.9-4.2) in head. Mouth large and oblique, length of upper jaw 2.0 (2.0-2.3) in head, maxilla width 7.3 (6.8-7.3) in head, maxillary roundly truncate and extending to rear edge of eye, with small supplemental bone, only visible through dissection.

Dorsal fin XI, 16 ~ 17; anal fin III, 8-9; pectoral fin 17–18; pelvic fin I, 5; caudal fin 16-19; lateral-line scales 71-86; lateral scale series 137-163; gill rakers 9-11+14-15; head length 35.3-38.5% SL; eye diameter 5.2-5.7% SL, preorbital length 7.1-7.6% SL and depth 4.3-5.7% SL, interorbital width 7.9-8.6% SL; snout length 8.3-9.4% SL, maxilla width 4.9-5.3% SL, length of upper jaw 15.8-18.9% SL, lower-jaw length 10.1-13.6% SL; body compressed laterally, body depth 34.2-35.3% SL and width 14.9-16.7% SL; predorsal length 30.9-32.6% SL, dorsal-fin base 56.6-58.4% SL, first dorsal spine 4.9-7.2% SL, second dorsal spine 9.9-13.6% SL, longest dorsal spine (usually fourth spine) 10.4-15.5% SL, last dorsal spine 8.6-10.9% SL, longest dorsal ray 11.7-14.7% SL; pre-anal length 63.6-68.0% SL, anal-fin base 7.4-17.1% SL, first anal spine 3.5-4.2% SL, second anal spine 6.5-8.4% SL, third anal spine 8.1-10.2% SL and longest anal ray 14.9-19.2% SL; pectoral-fin length 17.7-19.2% SL; prepelvic length 31.9-36.6% SL, pelvic-fin length 17.4-19.6% SL, pelvic-spine length 9.1-11.1% SL; caudal-fin length 19.3-20.9% SL, caudal-peduncle length 17.1-20.9% SL and depth 10.4-11.5% SL (see Table 1).

#### Diagnosis

*Epinephelusrankini* can clearly be distinguished from most of its congers by the absence of bars and bands in head and body (vs. presence) and diagnosed from confusable species by the following combination of characteristics: head, body and fins black greyish-brown to chocolate with irregular white blotches (vs. pale brownish-grey with irregular and small white blotches above in *E.multinotatus*; dark blue or greyish-blue in *E.flavocaeruleus*; dark reddish-brown in *Epinephelusmarginatus* Lowe,1834 and *Epinephelusmorio* Valenciennes,1828; olive to reddish-brown with irregular and large pale spots and blotches in *Epinepheluserythrurus* Valenciennes, 1828) ; absence of small dark brown spots (vs. numerous small dark reddish-brown spots below or spread all over the body in *E.multinotatus*; black spots in *E.cyanopodus*); the lateral-line scales 71-86 (vs. 48-51 in *Epinephelusclippertonensis* Allen and Robertson 1999); lateral-scale series 137-162 (vs. 92-107 *E.erythrurus*).

##### Molecular diagnosis (CBB)

*Epinephelusrankini* is diagnosed by a combination of 10 nucleotide substitutions: COI 276 (A→G), COI 279 (C→T), COI 294 (A→G), COI 351 (T→C), COI 399 (A→G), COI 402 (T→C), COI 442 (A→G), COI 519 (A→G), COI 537 (T→C), COI 552 (T→C). In addition, *E.rankini* possess 24 nucleotide substitutions when compared to *E.multinotatus*: COI 105 (T→A), COI 198 (T→C), COI 270 (C→T), COI 273 (T→C), COI 276 (A→G), COI 279 (C→T), COI 294 (A→G), COI 297 (C→T), COI 351 (T→C), COI 366 (G→A), COI 399 (A→G), COI 402 (T→C), COI 442 (A→G), COI 444 (A→C), COI 468 (A→G), COI 484 (C→T), COI 519 (A→G), COI 537 (T→C), COI 552 (T→C), COI 561 (G→A), COI 582 (T→C), COI 603 (G→A), COI 618 (C→T), COI 633 (A→G).

##### Colouration in life

Black greyish-brown to chocolate with irregular pale white blotches on the head, body and fins, with the blotches on the front of the head, chest and fins smaller and not obvious and those on both the sides of body larger; the rear margins of the unpaired soft rays have an extremely narrow white edge (Fig. 2a, c, e). Individuals with a bluish body colour are occasionally seen in the natural environment. When individuals are stressed or frightened, the pale white blotches become larger and darker and are regularly arranged on the side of the body from the back to the abdomen.

##### Colour in preservation

Body grey to brown with pale white blotches remaining prominent or fading. The narrow white edge on the posterior margin of the unpaired soft rays also remains or fades (Fig. 2c, e). With prolonged storage time, the body colour gradually turns yellowish-brown and the blotches and the white edge of the unpaired soft rays fade (Fig. 1a).

##### Distribution

**Figure 3.**
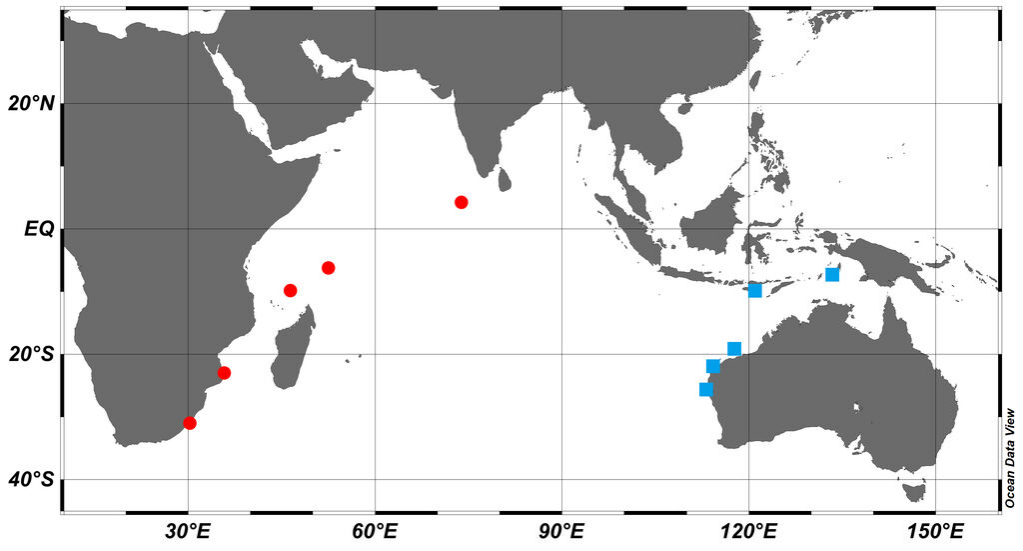
Map of the collection location of *Epinephelusrankini* (blue squares) and *Epinephelusmultinotatus* (red circles) examined in this study.

*Epinephelusrankini* is known from the Western Australian waters from the Abrolhos Islands northwards to Cape Leveque and south-eastern Indonesia (Fig. 3). *E.rankini* inhabits coral reefs and deeper offshore waters and can be found at depths up to 150 m (Rome and Newman 2010).

### Epinephelus multinotatus

Peters, 1876 (Figs. 1b and 2b, Table1)

111248B3-ACFA-59CB-A5BF-A65548C917FA

E666A249-758C-448C-B746-A888F43C5E21

#### Materials

- **Type status:** Holotype. **Taxon:** taxonID: ZMB 9452; scientificNameID: *Epinephelusmultinotatus*; kingdom: Animalia; phylum: Chordata; class: Actinopterygii; order: Perciformes; family: Epinephelidae; genus: Epinephelus; scientificNameAuthorship: (Peters 1876); **Location:** locality: Mauritius
- **Type status:** Paratype. **Occurrence:** recordNumber: ZMUA-epmul03-05; ZMUA-epmulD,ZMUA-epmulF, ZMUA-epmulG; individualCount: 6; **Taxon:** scientificNameID: *Epinephelusmultinotatus*; kingdom: Animalia; phylum: Chordata; class: Actinopterygii; order: Perciformes; family: Epinephelidae; genus: Epinephelus; **Location:** locality: Maldives; **Event:** year: 2020; **Record Level:** institutionCode: ZMUA; collectionCode: fish
- **Type status:** Paratype. **Occurrence:** recordNumber: ZMUA-epmul02; ZMUA-epmulE; individualCount: 2; **Taxon:** scientificNameID: *Epinephelusmultinotatus*; kingdom: Animalia; phylum: Chordata; class: Actinopterygii; order: Perciformes; family: Epinephelidae; genus: Epinephelus; **Location:** locality: South Africa; **Event:** year: 2019; **Record Level:** institutionCode: ZMUA; collectionCode: fish
- **Type status:** Paratype. **Occurrence:** recordNumber: SAIAB 77354, 80836; individualCount: 2; **Taxon:** scientificNameID: *Epinephelusmultinotatus*; kingdom: Animalia; phylum: Chordata; class: Actinopterygii; order: Perciformes; family: Epinephelidae; genus: Epinephelus; **Location:** locality: Seychelles; **Record Level:** institutionCode: SAIAB; collectionCode: fish
- **Type status:** Paratype. **Occurrence:** recordNumber: SAIAB 19541, 86850, 86834; individualCount: 3; **Taxon:** scientificNameID: *Epinephelusmultinotatus*; kingdom: Animalia; phylum: Chordata; class: Actinopterygii; order: Perciformes; family: Epinephelidae; genus: Epinephelus; **Location:** locality: Mozambique; **Record Level:** institutionCode: SAIAB; collectionCode: fish

#### Description

Head large and head length 2.2-2.7 in SL, orbit diameter 6.4-7.5 in head; interorbital broadly convex and width 4.1-5.2 in head. Snout length 3.6-4.6 in head. Mouth large and oblique, length of upper jaw 2.2-2.6 in head, maxilla width 7.1-8.6 in head, maxillary roundly truncate and extending to rear edge of eye, with small supplemental bone, only visible through dissection.

Dorsal fin XI, 15 ~ 17; anal fin III, 8; pectoral fin 16–20; pelvic fin I, 5; caudal fin 16-18; lateral-line scales 62-77; lateral scale series 130-151; gill rakers 9-11+15-17; head length 36.5-44.8% SL; eye diameter 5.3-6.1% SL, pre-orbital length 7.4-9.2% SL and depth 5.0-5.9% SL, interorbital width 7.4-8.9% SL; snout length 9.1-10.5% SL, maxilla width 5.0-5.3% SL, length of upper jaw 16.3-17.2% SL, lower-jaw length 11.3-12.0% SL; body compressed laterally, body depth 34.4-41.8% SL and width 14.4-19.8% SL; predorsal length 31.7-37.3% SL, dorsal-fin base 50.4-58.6% SL, first dorsal spine 3.5-5.7% SL, second dorsal spine 7.8-12.0% SL, longest dorsal spine (usually fourth spine) 11.3-14.0% SL, last dorsal spine 8.2-9.8% SL, longest dorsal ray 12.7-14.4% SL; pre-anal length 66.7-71.4% SL, anal-fin base 12.8-17.6% SL, first anal spine 2.6-3.5% SL, second anal spine 5.9-6.4% SL, third anal spine 7.8-10.3% SL and longest anal ray 15.9-16.0% SL; pectoral-fin length 17.0-19.3% SL; prepelvic length 30.9-35.7% SL, pelvic-fin length 16.3-19.4% SL, pelvic-spine length 9.7-10.3% SL; caudal-fin length 19.1-23.1% SL, caudal-peduncle length 16.0-20.6% SL and depth 11.3-11.6% SL (see Table 1).

#### Diagnosis

*Epinephelusmultinotatus* can clearly be distinguished from most of its congers by the absence of bars and bands in head and body (vs. presence), presence of numerous small dark reddish-brown spots (vs. absence or spots of other colour) and diagnosed from confusable species by the following combination of characteristics: head and body pale brownish-grey with irregular and small white blotches (vs. black greyish-brown to chocolate with irregular white blotches in *E.rankini*; dark blue or greyish-blue in *E.flavocaeruleus*; pale bluish-grey in *E.cyanopodus*; greyish-brown without blotches in *Epinephelusbontoides* Bleeker, 1855); absence of black saddle blotch (vs. presence of black saddle blotch in *Epinephelushowlandi* Gunther 1873), the lateral-line scales 62-77 (vs. 48-51 in *E.bontoides*), lateral-scale series 130-151 (vs. 88-109 in *Epinepheluscorallicola* Valenciennes, 1828).

##### Molecular diagnosis (CBB)

*Epinephelusmultinotatus* is diagnosed by a combination of 10 nucleotide substitutions: COI 105 (T→A), COI 198 (C→T), COI 270 (T→C), COI 273 (C→T), COI 366 (A→G), COI 484 (C→T), COI 561 (A→G), COI 582 (C→T), COI 618 (T→C), COI 633 (G→A).

##### Colouration in life

Pale brownish-grey with irregular and small white blotches above the head and body, and numerous small dark reddish-brown spots below the head and body, sometimes spread all over the body (Fig. 2b, dFig. 2d). The rear margins of the unpaired soft rays of some individuals have an extremely narrow white edge. When individuals are stressed or frightened, the black-brown striped blotches will appear behind the eyes.

##### Colour in preservation

Head and body pale brownish-grey with white blotches and small dark spots remaining prominent or fading. With prolonged storage time, the body colour gradually turns pale yellowish-brown and the blotches and spots fade (Figs 1b, 2d).

##### Distribution

*Epinephelusmultinotatus* is known from the Persian Gulf to southern Mozambique and also found in the island States (including Mauritius, Maldives, Seychelles, Madagascar, Réunion, Rodrigues and Chagos) (Fig. 3). Juveniles of *E.multinotatus* can be found on shallower inshore coral reefs and the adults can be found at depths up to 90 m.

## Analysis

**Figure 4.**
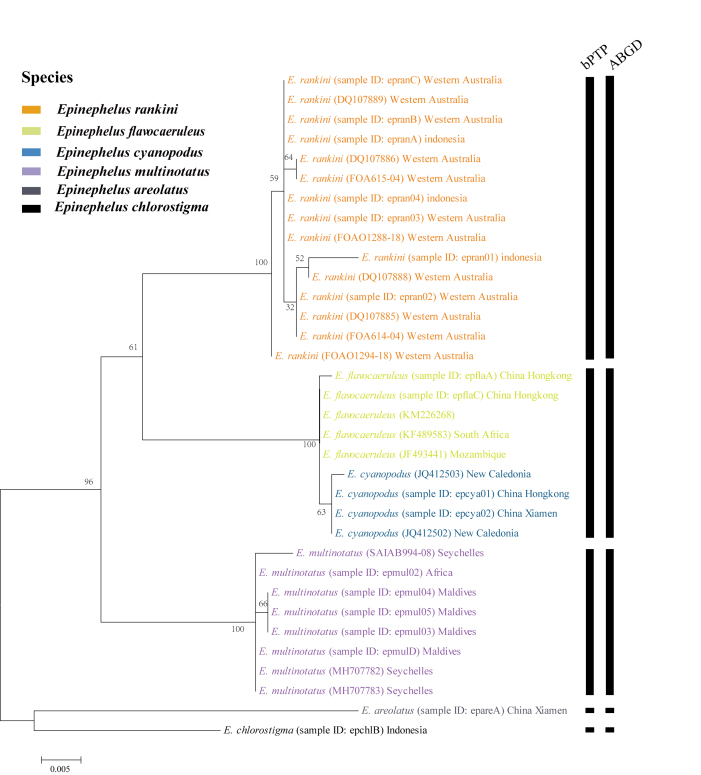
Species delimitation results of different methods of *Epinephelusrankini* and closely-related species, based on the COI sequences.

The mitochondrial *COI* gene sequences of *E.rankini* and closely-related species were sequenced or obtained from GenBank in this study. The intraspecific mean distance of *E.rankini* was 0.0023. The Kimura 2-parameter interspecific distances indicated that *E.rankini* differs from *E.multinotatus* by 0.0424, from *E.flavocaeruleus* by 0.0421, from *E.cyanopodus* by 0.0439, from *E.chlorostigma* by 0.0577 and from *E.areolatus* by 0.0753 (Table 2). Moreover, the phylogenetic tree showed that *E.rankini* formed a monophyletic clade and clustered with *E.flavocaeruleus* and *E.cyanopodus* on a large branch, while *E.multinotatus* clustered on a single branch (Fig. 4). In addition, a total of 34 specimens could be divided into five tentative species, based on bPTP analyses. *Epinephelusflavocaeruleus* and *E.cyanopodus* were grouped into the same tentative species, while the other four morphologically defined species, *E.multinotatus*, *E.rankini*, *E.chlorostigma* and *E.areolatus* were divided into four different tentative species (Fig. 4, Suppl. material 4). A similar result was obtained for the ABGD analyses; the specimens of *E.flavocaeruleus* and *E.cyanopodus* clustered into the same group (group 3) and the specimens of *E.rankini* and *E.multinotatus* clustered into group 1 and group 2, respectively (P = 0.001668–0.035938; Fig. 4, Suppl. material 5).

## Discussion

*Epinephelusmultinotatus* (type locality: Mauritius) is a western Indian Ocean species with a recorded maximum total length (TL) of 100 cm and was previously reported to be distributed from the Persian Gulf to southern Mozambique and eastwards to Western Australia (Craig et al. 2011). This species is divided into three independent differentiated populations, based on colour pattern and scale counts: the western Indian Ocean population (east coast of Africa, Comoros, Madagascar, Seychelles, Mauritius, Reunion, St. Brandon and the Chagos Archipelago), Persian Gulf and Gulf of Oman population, and Western Australian population (Heemstra and Randall 1993). The last population is *E.rankini*, reported to be distributed only in Western Australian waters, has been considered a synonym of *E.multinotatus* since 1987 due to similar morphological characteristics and white blotches (Polovina and Ralston 1987, Hutchins and Smith 1991). In this study, we re-examined specimens of both *E.rankini* and *E.multinotatus* and confirmed that both species are valid species belonging to the family Epinephelidae, which can be identified by morphological and molecular analyses.

**Morphological comparison**: Previous studies have shown that the western Indian Ocean population of white-blotched grouper has brown spots on the body and head (Heemstra and Randall 1993, M.R.C. 2003), the Persian Gulf and Gulf of Oman population usually has spots on the abdomen (Psoadakis et al. 2015) and the Western Australian population has no dark spots on the head and body (Whitley 1945). We also found significant differences in body colour patterns between *E.rankini* and *E.multinotatus* by re-examining the specimens: the head, body and fins of *E.rankini* are black greyish-brown to chocolate with irregular white blotches, but no small dark brown spots (Fig. 2a, c, e), while the head and body of *E.multinotatus* are pale brownish-grey with irregular and small white blotches above and numerous small dark brown spots below, sometimes spread all over the body (Fig. 2b, d). In addition to the colour patterns, we found that *E.rankini* could also be differentiated from *E.multinotatus* by its caudal fin shape [that in *E.rankini* slightly emarginate, while that in *E.multinotatus* truncate to slightly convex] (Fig. 1; Fig. 2a, b).

However, the population of *E.multinotatus*, distributed in the Gulf of Oman, is also similar to that of *E.rankini* in terms of caudal fin shape. It was first named *Epinephelusjayakari* (Boulenger, 1889), but encountered the same fate as *E.rankini*, becoming a synonym of *E.multinotatus* from the Arabian Gulf and Oman region (Heemstra and Randall 1993, Froese and Pauly 2022), although some researchers consider it to be a valid species, based on existing records (Psoadakis et al. 2015). Unfortunately, we did not collect samples from the Gulf of Oman area. However, according to previous studies (Heemstra and Randall 1993, M.R.C. 2003, Psoadakis et al. 2015), regardless of whether *E.multinotatus* is distributed in the Gulf of Oman or the western Indian Ocean, *E.rankini* and *E.multinotatus* can be distinguished based on differences in body colour patterns and are easier to distinguish when the specimens are fresh.

**Genetics**: The interspecific mean distance between *E.rankini* and *E.multinotatus* was 0.0424, which was greater than the interspecific mean distance (0.0421) between *E.rankini* and *E.flavocaeruleus*, that (0.0260) between *Epinephelusbruneus* (Bloch 1793) and *E.moara* (Liu et al. 2013) and that (0.0321) between *Epinephelusgabriellae* (Randall and Heemstra) and *E.geoffroyi* (Wu et al. 2020), indicating that *E.rankini* is a valid species. In addition, the ML tree, CBB, ABGD and bPTP analyses also strongly support this conclusion (Fig. 4). The interspecific distance between *E.flavocaeruleus* and *E.cyanopodus* is only 0.0023, much smaller than the average intraspecific distance (COI: 0.0034), which is consistent with the results obtained by Qu et al. (2018). In addition, the ML tree, bPTP and ABGD analyses also clustered *E.flavocaeruleus* and *E.cyanopodus* into the same group, supporting that they are possible synonyms. *Epinephelusflavocaeruleus* and *E.cyanopodus* are considered two valid species due to differences in the body colour pattern and geographical distribution (Heemstra and Randall 1993, Craig et al. 2011). However, the same individual may have different colour patterns in different living environments of groupers, so more systematic studies are needed to clarify their taxonomic relationships.

**Ecological notes**: We observed that *E.rankini* and *E.multinotatus* exhibit different appearances when stressed or startled. The blotches of *E.rankini* become larger and regularly arranged on the side of the body from the back to the abdomen, while white blotches of *E.multinotatus* remain unchanged in body, but there are black-brown striped blotches behind the eyes. In addition, *E.rankini* inhabits coral reefs and deeper offshore waters and can be found at depths up to 150 m (Rome and Newman 2010), while *E.multinotatus* can be found in shallower depths of 90 m and juveniles are found on shallower inshore coral reefs (M.R.C. 2003). *E.rankini* was initially described only in Western Australian seas from the Abrolhos Islands northwards to Cape Leveque (Rome and Newman 2010), but we also collected some samples in the southern sea area of the Indonesia Archipelago (Fig. 3). Since Western Australia and Indonesia are not far apart and there is no geographic barrier between them, the existence of such a migration is acceptable. Of course, more samples are still needed to confirm this distribution.

### Conclusion

We consider *E.rankini* to be a valid species based on morphological and molecular analysis. It can be distinguished from *E.multinotatus* by the following combination of characters: body black greyish-brown to chocolate with irregular whitish blotches, without small dark brown spots and slightly emarginate caudal fin. Molecular analysis also strongly supports *E.rankini* as a distinct species. In addition, the sample collection results indicated that *E.rankini* was known to be distributed from the Abrolhos Islands of Western Australia to south-eastern Indonesia. *E.rankini* and *E.multinotatus* should be recorded separately in the future to assess the stock levels for sustainable fisheries.

## Supplementary Material

XML Treatment for Epinephelus rankini

XML Treatment for Epinephelus multinotatus

24B0AFA2-5CAB-53FF-8834-60C2F23481CB 10.3897/BDJ.10.e90472.suppl1 Supplementary material 1 List of examined material for morphology Data type Examined material for morphology File: oo_737137.docx https://binary.pensoft.net/file/737137 Xiaoying Cao, Haohao Wu, Haoran Zhang, Lina Wu, Shaoxiong Ding

E21CDFBF-FE35-5883-AEA6-FC4C30D9DDD4 10.3897/BDJ.10.e90472.suppl2 Supplementary material 2 Genetic Samples information Data type Genetic Samples information File: oo_750328.docx https://binary.pensoft.net/file/750328 Xiaoying Cao, Haohao Wu, Haoran Zhang, Lina Wu, Shaoxiong Ding

A1028383-F839-5032-9991-AC1651BC5A22 10.3897/BDJ.10.e90472.suppl3 Supplementary material 3 Bayesian Inference phylogenetic tree. Data type the results of CBB analysis File: oo_752708.docx https://binary.pensoft.net/file/752708 Xiaoying Cao, Haohao Wu, Haoran Zhang, Lina Wu, Shaoxiong Ding

D1680486-FBB0-5FEC-A05F-344264419462 10.3897/BDJ.10.e90472.suppl4 Supplementary material 4 Detailed result of the bPTP analysis Data type Sequence analysis results File: oo_752703.docx https://binary.pensoft.net/file/752703 Xiaoying Cao, Haohao Wu, Haoran Zhang, Lina Wu, Shaoxiong Ding

D4BD3EF5-DE76-55EA-8368-0AE963CE5978 10.3897/BDJ.10.e90472.suppl5 Supplementary material 5 Detailed result of the ABGD analysis (*P* = 0.001668 – 0.035938; Barcode gap distance = 0.023) Data type Sequence analysis results File: oo_752706.docx https://binary.pensoft.net/file/752706 Xiaoying Cao, Haohao Wu, Haoran Zhang, Lina Wu, Shaoxiong Ding

## Figures and Tables

**Figure 1a.**
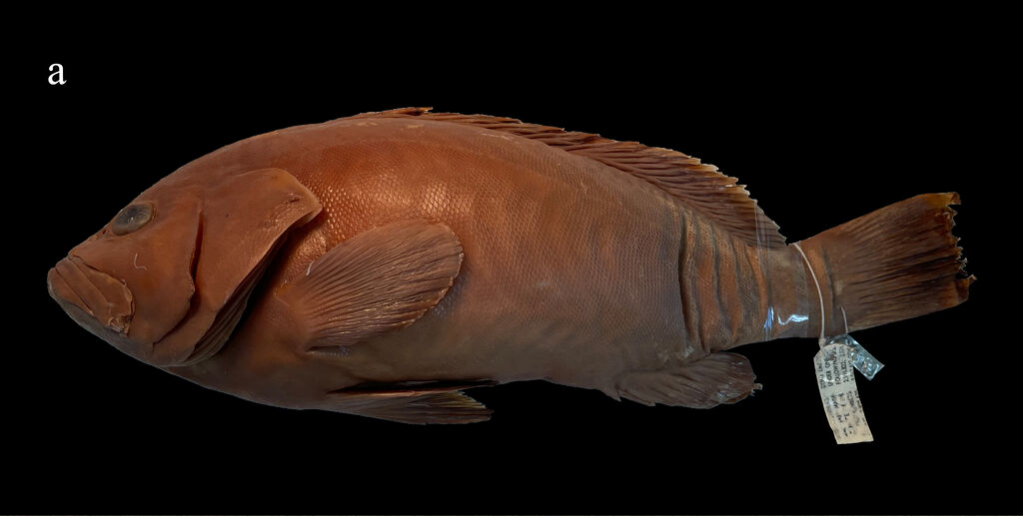
*Epinephelusrankini*, holotype (WAM P.2847-001), 330 mm, photographed by Juntong Hu

**Figure 1b.**
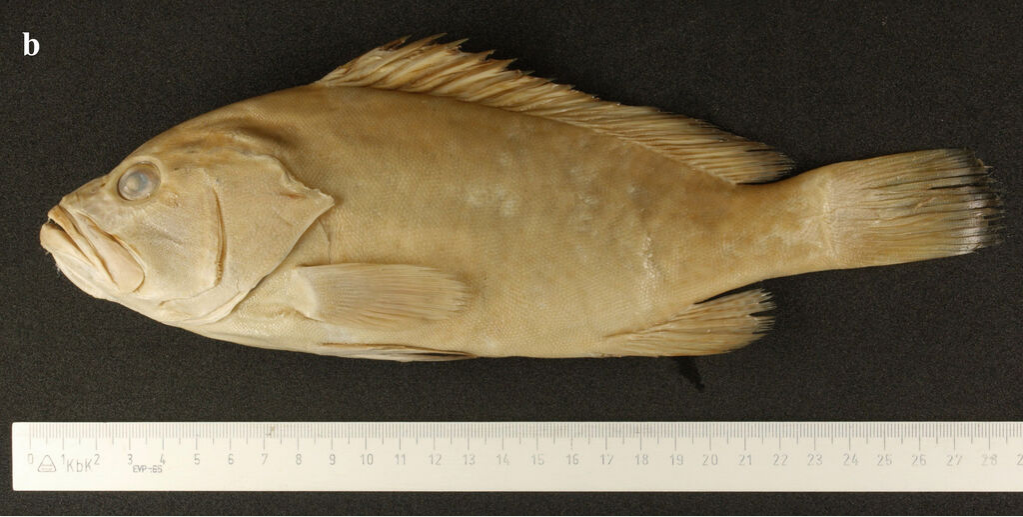
*Epinephelusmultinotatus*, holotype (ZMB 9452), 231 mm, photographed by Edda Aßel

**Figure 2a.**
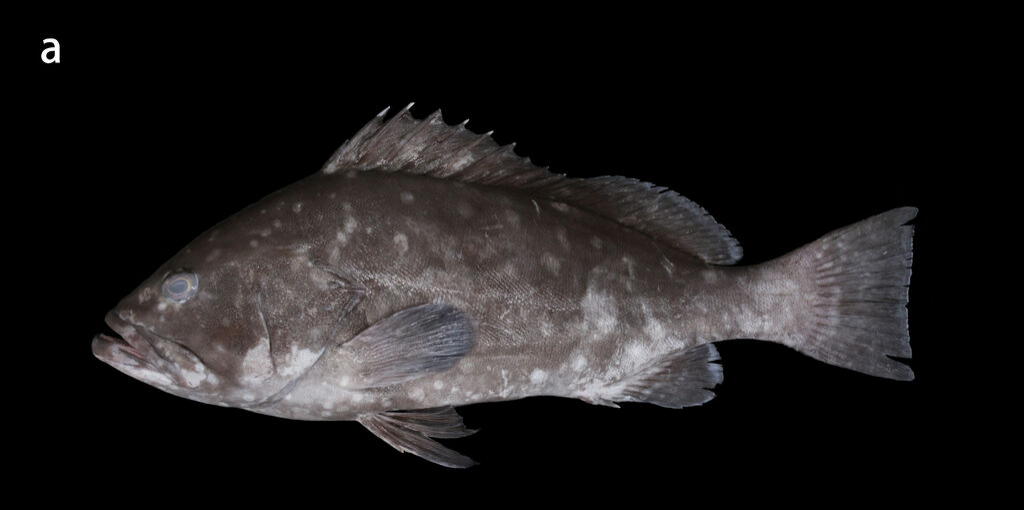
fresh specimens of *E.rankini*, ZMUA-epran02, 368 mm SL, caught in the waters of Thevenard Island, Western Australia

**Figure 2b.**
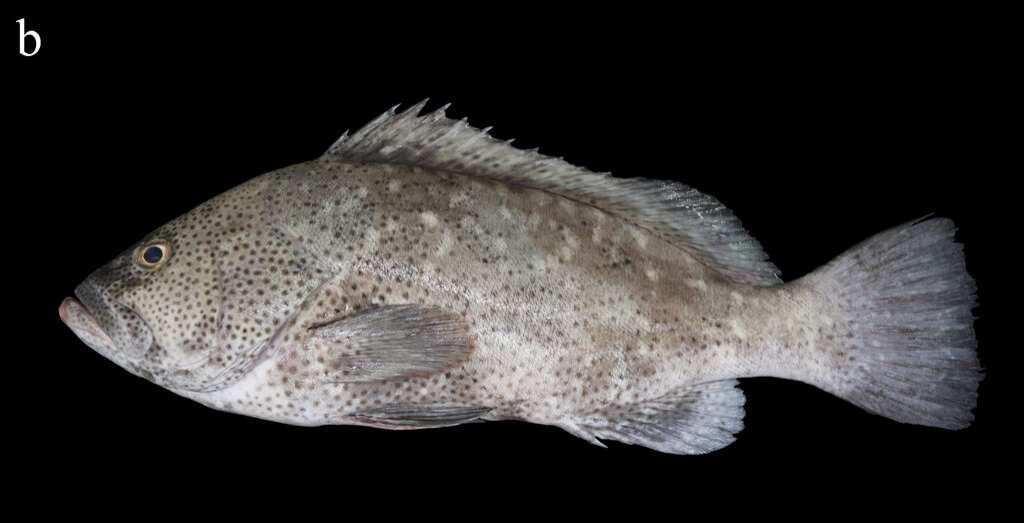
fresh specimens of *E.multinotatus*, ZMUA-epmul02, 308.9 mm SL, caught in Africa

**Figure 2c.**
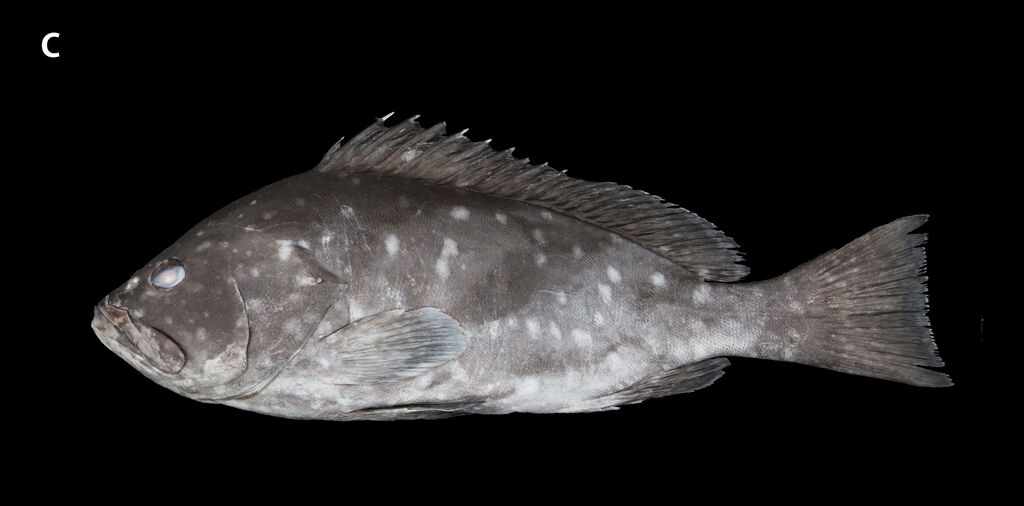
preserved specimens of *E.rankini*, ZMUA-epran02, 368 mm SL

**Figure 2d.**
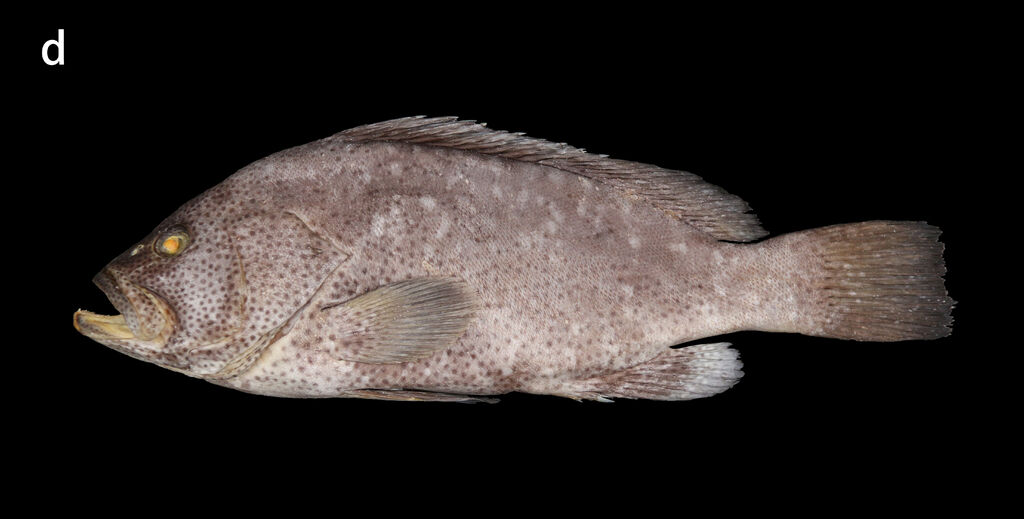
preserved specimens of *E.multinotatus*, ZMUA-epmul02, 308.9 mm SL

**Figure 2e.**
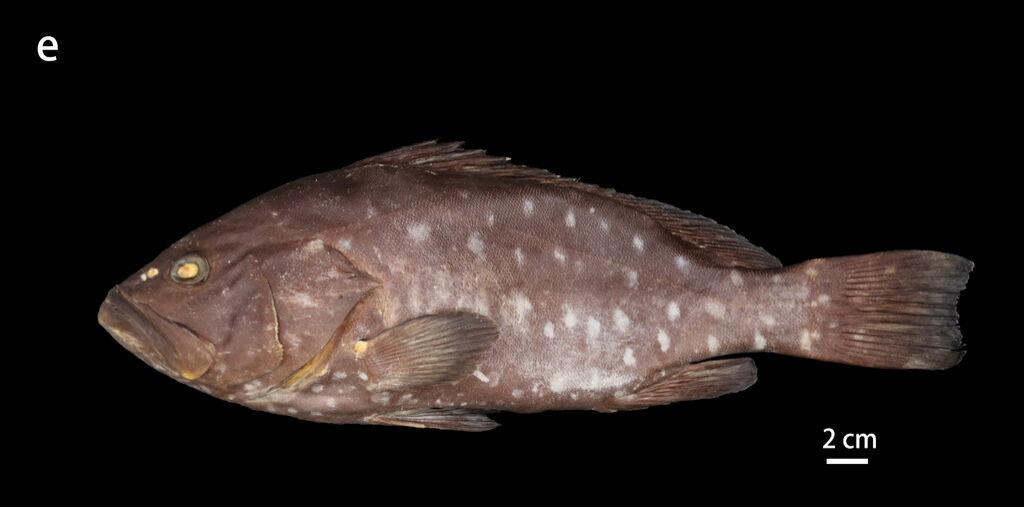
preserved specimens of *E.rankini*, ZMUA-epran01, 357 mm SL

**Table 1.** Meristics and measurements of *E.rankini* and *E.multinotatus*

	*Epinephelusrankini*		*Epinephelusmultinotatus*	
	Holotype WAM P.2847-001	Range for specimens	Holotype ZMB 9452	Range for specimens
Total length (mm)	330	305-456	282	286-713
Standard length (mm)	265	240-385	231	231-645
Dorsal-fin ray count	XI, 16	XI, 16-17	XI, 16	XI, 15-17
Anal-fin ray count	III, 8	III, 8-9	III,8	III, 8
Pelvic-fin ray count	I-5	I-5	-	I-5
Pectoral-fin ray count	18	17-18	16	16-20
Caudal-fin ray count	18	16-19	18	16-18
Lateral line	-	71-86	-	62-77
Lateral line series	163	137-163	-	130-151
Gill rakers	9+15	9-11+14-15	-	9-11+15-17
% of S				
Head length	37.7	35.3-38.5	38.1	36.5-44.8
Eye diameter	5.7	5.2-5.7	6.1	5.3-6.1
Pre-orbital length	7.5	7.1-7.6	-	7.4-9.2
Pre-orbital depth	4.5	4.3-5.7	-	5.0-5.9
Interorbital width	8.5	7.9-8.6	-	7.4-8.9
Snout length	9.4	8.3-9.4	9.1	9.1-10.5
Maxilla width	5.3	4.9-5.3	-	5.0-5.3
Length of upper jaw	17.0	15.8-18.9	-	16.3-17.2
Lower-jaw length	13.6	10.1-13.6	-	11.3-12.0
Body depth	34.7	34.2-35.3	-	34.4-41.8
Body width	22.6	14.9-16.7	-	14.4-19.8
Predorsal length	30.9	30.9-32.6	34.6	31.7-37.3
Dorsal-fin base	56.6	56.6-58.4	-	50.4-58.6
First dorsal spine	7.2	4.9-7.2	-	3.5-5.7
Second dorsal spine	13.6	9.9-13.6	-	7.8-12
Longest dorsal spine	14.7	10.4-15.5	-	11.3-14.0
Last dorsal spine	10.9	8.6-10.9	-	8.2-9.8
Longest dorsal ray	14.3	11.7-14.7	-	12.7-14.4
Pre-anal length	66.8	63.6-68.0	71.4	66.7-71.4
Anal-fin base	17	7.4-17.1	-	12.8-17.6
First anal spine	4.2	3.5-4.2	-	2.6-3.5
Second anal spine	7.9	6.5-8.4	-	5.9-6.4
Third anal spine	10.2	8.1-10.2	-	7.8-10.3
Longest anal ray	19.2	14.9-19.2	-	15.9-16.0
Pectoral-fin length	17.7	17.7-19.2	-	17.0-19.3
Prepelvic length	36.2	31.9-36.6	35.5	30.9-35.7
Pelvic-fin length	18.9	17.4-19.6	-	16.3-19.4
Pelvic-spine length	11.3	9.1-11.3	-	9.7-10.3
Caudal-peduncle length	19.6	17.1-20.9	16.5	16.0.-20.6
Caudal-peduncle depth	11.3	10.4-11.5	-	11.3-11.6
Caudal-fin length	19.6	19.3-20.9	22.1	19.1-23.1

**Table 2.** Analysis of intraspecific and interspecific mean distances (K2P model), based on COI sequences between *E.rankini* and close-related species; interspecific distances (lower left in diagonal)；Standard errors (upper right in diagonal); IMD: intraspecific mean distance; SE: standard error.

**Group**		**Species**	**N**	**IMD**	**SE**	**Interspecific Mean Distance**					
						**1**	**2**	**3**	**4**	**5**	**6**
1	*E.rankini*		15	0.0023	0.0009	-	0.0078	0.0079	0.0081	0.0097	0.0112
2	*E.multinotatus*		8	0.0020	0.0010	0.0424	-	0.0079	0.0081	0.0098	0.0103
3	*E.flavocaeruleus*		5	0.0006	0.0006	0.0421	0.0437	-	0.0016	0.0107	0.0110
4	*E.cyanopodus*		4	0.0008	0.0007	0.0439	0.0455	0.0023	-	0.0109	0.0113
5	*E.chlorostigma*		1	-	-	0.0577	0.0536	0.0650	0.0669	-	0.0109
6	*E.areolatus*		1	-	-	0.0753	0.0689	0.0735	0.0753	0.0626	
