# Supplementary material for: Epinephelusrankini Whitley, 1945, a valid species of grouper (Teleostei, Perciformes, Epinephelidae) from Western Australia and southeast Indonesia
Source: Biodivers Data J. 2022 Oct 14;10:e90472. doi: 10.3897/BDJ.10.e90472 (PMC9836616; doi:10.3897/BDJ.10.e90472)
Supplement: Supplementary material 1 — List of examined material for morphology [file bdj-10-e90472-s001.docx]

**Table S1 List of examined material for morphology**

***Epinephelus multinotatus******:***

MAURITIUS: ZMB 9452, 231 mm (holotype of *Serranus multinotatus*); SAIAB 69813, 240 mm;

MALDIVES: ZMUA-epmul03, 268 mm; ZMUA-epmul04, 282 mm; ZMUA-epmul05, 287 mm; ZMUA-epmulD, 634mm; ZMUA-epmulF, 455 mm; ZMUA-epmulG, 306mm; MOZAMBIQUE: SAIAB 19541, 519 mm; SAIAB 86850, 550 mm; SAIAB 86834, 645 mm; SEYCHELLES: SAIAB 77354, 333 mm; SAIAB 80836, 550 mm. SOUTH AFRICA: ZMUA-epmul02, 309 mm; ZMUA-epmulE, 380mm;

***Epinephelus*** ***rankini:***

AUSTRALIA: WAM P.2847-001, 330mm SL (holotype); ZMUA-epran02, 368mm; ZMUA-epran03, 385mm; ZMUA-epran03, 240mm; ZMUA-epranB, 283mm; ZMUA-epranC, 326mm; INDONESIA: ZMUA-epran01, 357 mm; ZMUA-epranA, 268mm.
