# Supplementary material for: Epinephelusrankini Whitley, 1945, a valid species of grouper (Teleostei, Perciformes, Epinephelidae) from Western Australia and southeast Indonesia
Source: Biodivers Data J. 2022 Oct 14;10:e90472. doi: 10.3897/BDJ.10.e90472 (PMC9836616; doi:10.3897/BDJ.10.e90472)
Supplement: Supplementary material 2 — Genetic Samples information [file bdj-10-e90472-s002.docx]

**Table S2.** **Genetic Samples information**

| Species | Individual Number | Samples ID | Collection Date | Collection Locality | GenBank/BOLD Accession Numbers | References |
| --- | --- | --- | --- | --- | --- | --- |
|  |  |  |  |  | *COI* |  |
| *Epinephelus rankini* | 15 | epran01 | Apr-2019 | Indonesia | ON117789 | This Research |
|  |  | epran02 | Nov-2021 | Western Australia | ON117790 | This Research |
|  |  | epran03 | Nov-2021 | Western Australia | ON117791 | This Research |
|  |  | epran04 | Dec-2022 | Western Australia | ON117792 | This Research |
|  |  | epranA | 2006 | Indonesia | ON117793 | This Research |
|  |  | epranB | Aug-2019 | Western Australia | ON117793 | This Research |
|  |  | epranC | Aug-2019 | Western Australia | ON117795 | This Research |
|  |  | BW-A14521 /CSIRO H.8231-01 | - | Western Australia | FOAO1288-18 | - |
|  |  | BW-A614 /CSIRO H.3834 -01 | - | Western Australia | FOA614-04 | - |
|  |  | BW-A615 | - | Western Australia | FOA615-04 | - |
|  |  | BW-A14529 | - | Western Australia | FOAO1294-18 | - |
|  |  | DQ107885 | 11-Nov-1994 | Western Australia | DQ107885 | - |
|  |  | DQ107886 | 11-Nov-1994 | Western Australia | DQ107886 | - |
|  |  | DQ107888 | 31-Oct-1994 | Western Australia | DQ107888 | - |
|  |  | DQ107889 | 31-Oct-1994  - | Western Australia | DQ107889 | - |
| *E. multinotatus* | 8 | MH707782 | - | Seychelles | MH707782 | - |
|  |  | MH707783 | - | Seychelles | MH707783 | - |
|  |  | epmul02 | Oct-2019 | South Africa | ON117796 | This Research |
|  |  | epmul03 | Jun-2020 | Maldives | ON117796 | This Research |
|  |  | epmul04 | Jun-2020 | Maldives | ON117798 | This Research |
|  |  | epmul05 | Jun-2020 | Maldives | ON117799 | This Research |
|  |  | epmulD | Oct-2019 | Maldives | ON117800 | This Research |
|  |  | SAIAB 80836/ TAN 0807-0685 | - | Seychelles | TAN 0807-0685 | - |
| *E. flavocaeruleus* | 5 | KM226268 | - | - | KM226268 | - |
|  |  | KF489583 | 08-Dec-2010 | South Africa | KF489583 | - |
|  |  | JF493441 | 01-May-2008 | Mozambique | JF493441 | - |
|  |  | epflaA | 2005 | Hong Kong, China | MF185511 | Qu et al. 2018 |
|  |  | epflaC | Dec-2016 | Shenzheng,China | ON117801 | This Research |
| *E. cyanopodus* | 4 | JQ412503 | 28-Sep-2010 | New Caledonia | JQ412503 | - |
|  |  | JQ412502 | 25-Oct-2005 | New Caledonia | JQ412502 | - |
|  |  | epcya01 | Jan-2012 | Hong Kong, China | MF185487 | Qu et al. 2018 |
|  |  | epcya02 | Jan-2015 | Xiamen,Chian | MF185488 | Qu et al. 2018 |
| *E. areolatus* | 1 | epareA | 2005 | Xiamen,Chian | ON117802 | This Research |
| *E. chlorostigma* | 1 | epchlB | 2006 | Indonesia | MK729757 | Wu et al. 2020 |

**References**

Qu M, Tang W, Liu Q, Wang D, Ding SJ. 2018. Genetic diversity within grouper species and a method for interspecific hybrid identification using DNA barcoding and ryr3 marker. Molecular phylogenetics 121: 46-51.

Wu H, Qu M, Lin H, Tang W, Ding S. 2020. *Epinephelus tankahkeei*, a new species of grouper (teleostei, perciformes, epinephelidae) from the south china sea. J ZooKeys 933: 125-137.
