# Supplementary material for: Epinephelusrankini Whitley, 1945, a valid species of grouper (Teleostei, Perciformes, Epinephelidae) from Western Australia and southeast Indonesia
Source: Biodivers Data J. 2022 Oct 14;10:e90472. doi: 10.3897/BDJ.10.e90472 (PMC9836616; doi:10.3897/BDJ.10.e90472)
Supplement: Supplementary material 3 — Bayesian Inference phylogenetic tree. [file bdj-10-e90472-s003.docx]

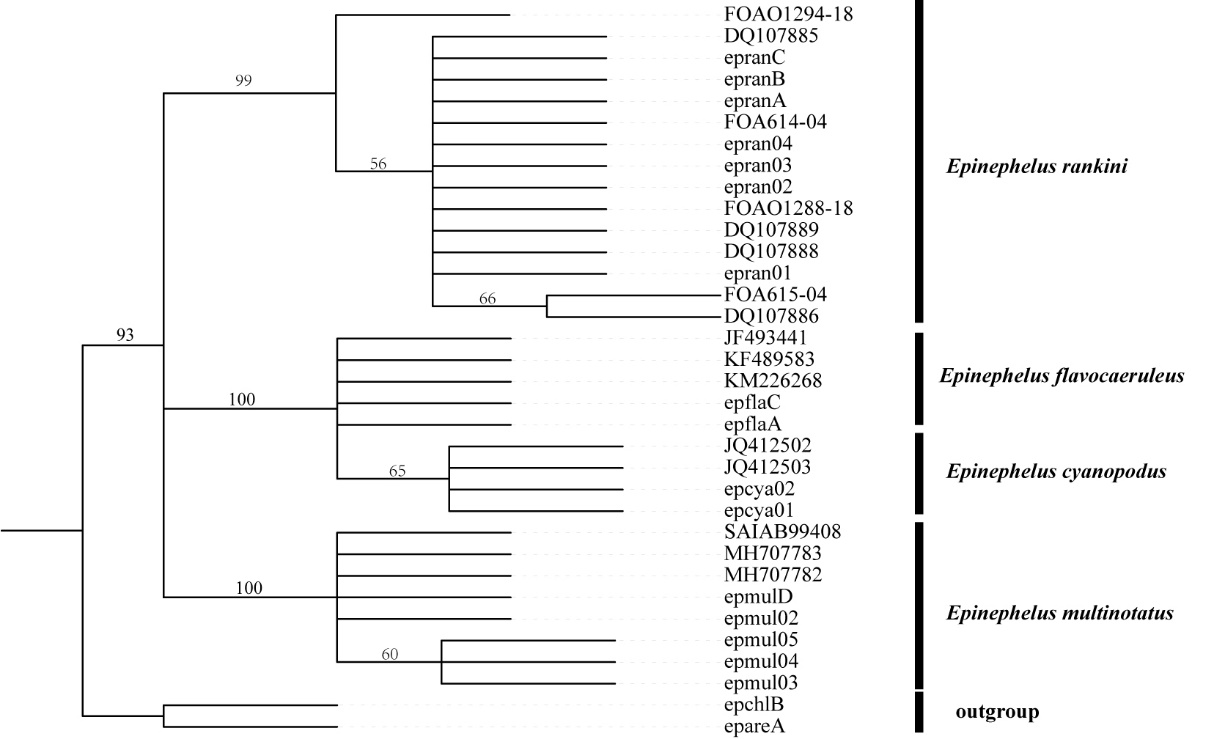


Table S3 Bayesian Inference phylogenetic tree. Numbers above branches are posterior probability values.
