# Supplementary material for: Epinephelusrankini Whitley, 1945, a valid species of grouper (Teleostei, Perciformes, Epinephelidae) from Western Australia and southeast Indonesia
Source: Biodivers Data J. 2022 Oct 14;10:e90472. doi: 10.3897/BDJ.10.e90472 (PMC9836616; doi:10.3897/BDJ.10.e90472)
Supplement: Supplementary material 4 — Detailed result of the bPTP analysis [file bdj-10-e90472-s004.docx]

**Table S4 Detailed result of the bPTP analysis**

| Marker | bPTP_Speices | Support | Number of haplotype | Morphospecies |
| --- | --- | --- | --- | --- |
| COI | Species 1 | 0.757 | 1 | E. areolatus |
|  | Species 2 | 0. 757 | 1 | *E. chlorostigma* |
|  | Species 3 | 0.174 | 6 | *E. rankini* |
|  | Species 4 | 0.306 | 4 | E. flavocaeruleus & E. cyanopodus |
|  | Species 5 | 0.155 | 3 | E. multinotatus |
