# Supplementary material for: Epinephelusrankini Whitley, 1945, a valid species of grouper (Teleostei, Perciformes, Epinephelidae) from Western Australia and southeast Indonesia
Source: Biodivers Data J. 2022 Oct 14;10:e90472. doi: 10.3897/BDJ.10.e90472 (PMC9836616; doi:10.3897/BDJ.10.e90472)
Supplement: Supplementary material 5 — Detailed result of the ABGD analysis (P = 0.001668 – 0.035938; Barcode gap distance = 0.023) [file bdj-10-e90472-s005.docx]

**Table S5 Detailed result of the ABGD analysis (*P* =** **0.001668 – 0.035938; Barcode gap distance = 0.023)**

| Marker | Group | Morphospecies | Number |
| --- | --- | --- | --- |
| COI | Group1 | *E. rankini* | 15 |
|  | Group2 | E. multinotatus | 8 |
|  | Group3 | E. flavocaeruleus & E. cyanopodus | 9 |
|  | Group4 | E. areolatus | 1 |
|  | Group5 | *E. chlorostigma* | 1 |
